# Linking Microbial Enzymatic Activities and Functional Diversity of Soil around Earthworm Burrows and Casts

**DOI:** 10.3389/fmicb.2016.01361

**Published:** 2016-08-30

**Authors:** Jerzy Lipiec, Magdalena Frąc, Małgorzata Brzezińska, Marcin Turski, Karolina Oszust

**Affiliations:** Institute of Agrophysics, Polish Academy of SciencesLublin, Poland

**Keywords:** pear orchard, earthworm-built structures, enzymes, community-level physiological profile, microbial functional diversity

## Abstract

The aim of this work was to evaluate the effect of earthworms (Lumbricidae) on the enzymatic activity and microbial functional diversity in the burrow system [burrow wall (BW) 0–3 mm, transitional zone (TZ) 3–7 mm, bulk soil (BS) > 20 mm from the BW] and cast aggregates of a loess soil under a pear orchard. The dehydrogenase, β-glucosidase, protease, alkaline phosphomonoesterase, and acid phosphomonoesterase enzymes were assessed using standard methods. The functional diversity (catabolic potential) was assessed using the Average Well Color Development and Richness Index following the community level physiological profiling from Biolog Eco Plates. All measurements were done using soil from each compartment immediately after *in situ* sampling in spring. The enzymatic activites including dehydrogenase, protease, β-glucosidase and alkaline phosphomonoesterase were appreciably greater in the BW or casts than in BS and TZ. Conversely, acid phosphomonoesterase had the largest value in the BS. Average Well Color Development in both the TZ and the BS (0.98–0.94 A_590 nm_) were more than eight times higher than in the BWs and casts. The lowest richness index in the BS (15 utilized substrates) increased by 86–113% in all the other compartments. The PC1 in principal component analysis mainly differentiated the BWs and the TZ. Utilization of all substrate categories was the lowest in the BS. The PC2 differentiated the casts from the other compartments. The enhanced activity of a majority of the enzymes and increased microbial functional diversity in most earthworm-influenced compartments make the soils less vulnerable to degradation and thus increases the stability of ecologically relevant processes in the orchard ecosystem.

## Introduction

Earthworms are considered as the most important soil ecosystem engineers in soils through building of burrows and cast production (Jégou et al., 2001; Lavelle, 2011). Presence of burrows enhances water infiltration (Strudley et al., 2008; Alaoui et al., 2011) and allow preferential flow, decrease runoff and water erosion (Holz et al., 2015), influence the movement of water and solutes from channels to soil matrix and vice versa (Jégou et al., 2001; Lipiec et al., 2015), and improve root penetrability (Whalley and Dexter, 1994; Głąb, 2013). Earthworm casts during aging become strong and water stable aggregates and improve soil aggregation more than plant roots (e.g., Blanchart et al., 2004b). Stable soil aggregation is essential for water and gas transfer (Capowiez et al., 2006; Gerke, 2006) as well as soil protection against crusting, erosion and compaction (Horn, 2004; Alaoui et al., 2011). Furthermore, earthworms contribute to removal of organic contaminants from soil (Dallinger and Horn, 2014) contained in fungicides, and facilitate chelation of metal ions (Lavelle et al., 1995; Cai et al., 2002).

Earthworm burrows provide a habitat for other invertebrates, e.g., nematodes (Görres et al., 2001; Andriuzzi et al., 2016) and arthropods (Boivin et al., 2006), which jointly provide drilosphere with organic carbon. The term drilosphere is defined as the soil region around burrows (within several millimeters) and represents an important microbial hotspot in soil (Bouché, 1975; Schrader et al., 2007). In the study by Stromberger et al. (2012), total C and labile (low molecular weight) C concentrations were greater in the drilosphere than in nearby bulk soil (BS) by 23 and 58%, respectively. The input of labile C and energy can affect the abundance of the microbial community and enzymatic activity in the drilosphere and casts built in soil (Jégou et al., 2001; Valchovski, 2011; Lipiec et al., 2015) and in vermicompost (Sen and Chandra, 2009; Kostecka and Pączka, 2011) leading to priming effect (Kuzyakov et al., 2000; Bundt et al., 2001). Also fresh earthworm cast aggregates (CAs) with a high C input stimulate microbial development (Zirbes et al., 2012). On the other hand the microorganisms in earthworm-built structures are an unavoidable constituent of earthworms’ natural diet (Pizl and Novakova, 2003) and thus the structures may affect microbial abundance and activity through direct trophic effects (Andriuzzi et al., 2016).

As indicated in recent a review by Kuzyakov and Blagodatskaya (2015) most studies have concentrated on microbial hotspots, created in the rhizosphere and detritusphere and only few results are available in other hotspots including earthworm drilospheres and casts, although they affect soil and ecosystem functioning and serve as proxies for organic matter degradation (Moorhead et al., 2012). Dehydrogenase is an intracellular enzyme that plays an essential role in the initial stages of oxidation of organic matter by transferring electrons from substrates to acceptors (Oszust et al., 2014). The other soil enzyme activities have been suggested as suitable indicators of soil quality because they are a measure of the soil microbial activity and therefore they are strictly related to the nutrient cycles and transformations and they rapidly may respond to the changes caused by both natural and anthropogenic factors (Frąc and Jezierska-Tys, 2011).

Therefore, in this study, we tested the hypothesis that alterations in the structure and organic carbon concentrations affect enzymatic activities including dehydrogenase, protease, β-glucosidase, alkaline phosphomonoesterase, acid phosphomonoesterase, and the microbial functional diversity (metabolic potential) in the drilosphere and casts made by earthworms in pear orchard field.

## Materials and Methods

### Site and Soil Sampling

The study was conducted at the experimental farm of the Lublin University of Life Sciences in Felin (51°15′N, 22°35′E), in the south-eastern part of Poland. The climate is moderately warm continental. The long-term annual mean temperature and precipitation at the experimental site are 7.4°C and 572 mm, respectively. The soil is a Haplic Luvisol (World Reference Base, 2014) derived from loess, over limestone with silt loam texture containing (in g kg^-1^) 660 sand (2–0.02 mm), 280 silt (0.02–0.002 mm), and 60 clay (<0.002 mm), and characterized by pH (H_2_O) 5.85, bulk density 1.33 Mg m^-3^ and particle density 2.61 Mg m^-3^ (Lipiec et al., 2012). The research area has a rather uniform textural composition of soils (Dobrzański and Zawadzki, 1951). The soil was sampled from a 50-year-old pear orchard with a permanent sward consisting of various species of grasses and legumes that were regularly mown in the inter-rows during growing seasons. The orchard is inhabited mostly by endogeics, or topsoil dwelling earthworms and anecics, or deep burrowing including subsoil-dwelling earthworms (Bouché, 1977).

The endogeic *Aporrectodea caliginosa* (Savigny, 1826) and *Allolobophora chlorotica* (Savigny, 1826) and the anecic *Lumbricus terrestris* (Linnaeus, 1758) are widespread earthworm species in the orchard. We observed that the burrows 4–7 mm in diameter predominate in the orchard, and therefore such burrows were chosen for studying.

We used the following compartments: the burrow wall (BW) up to 3 mm from the BW; the transitional zone (TZ) 3–7 mm from the BW; the BS, situated at least 20 mm from the BW, and the earthworm CA. These compartments were similar to those used in Jégou et al. (2001). To obtain sufficient quantity of soil for determination of the microbial enzymatic activities and functional diversity about 95 undisturbed soil samples containing burrows were taken from the pear orchard field (2400 m^2^). At least 1.0 m between burrows was supposed enough for independence of replicates. *In situ* soil samples from BW and TZ were collected by scraping the BWs. Soil from all compartments was taken from the surface and BS at the upper 10 cm from 18 locations and mixed into representative sample. Then three replicates from the representative sample of the fresh soil were sieved through a 2-mm mesh and weighted and tested. All samples were taken in spring 2011 when the soil was moist. We focused on topsoil where earthworms largely affect biological activity (e.g., Simonsen et al., 2010). The casts were collected from soil surface.

### Enzymatic Analysis

Dehydrogenase activity was determined according to the method of Casida et al. (1964) with the use of TTC (2,3,5-triphenyl tetrazolium chloride). After the incubation, the triphenyl formazan formed was extracted with ethanol and assayed at 485 nm. Protease activity was determined with the method of Ladd and Butler (1972) modified by Alef and Nannipieri (1995), with sodium caseinate as the substrate. The activity of the enzyme was assayed spectrophotometrically at a wavelength of 578 nm in Tris-HCl buffer with pH 8.1. The activity of acid and alkaline phosphomonoesterase was assayed with the method of Tabatabai and Bremner (1969) using *p*-nitrophenol (PNP) in TRIS-HCl buffer with pH 6.5 for acid phosphomonoesterase and pH 11 for alkaline phosphatase. The enzymatic activity was determined colorimetrically at a wavelength of 400 nm. The β-glucosidase activity was determined according to Alef and Nannipieri (1995) with the method based on determination of released PNP after the incubation of soil with a *p*-nitrophenyl glucoside (PNG) solution for 1 h at 37°C. The enzymatic activity was determined colorimetrically at wavelength of 400 nm.

### Community-Level Physiological Profiles

The potential ability of the microbial community to utilize the selected carbon sources was assessed by determining the community-level physiological profiles (CLPPs) with the Biolog Eco microplate identification system (Biolog, USA). The EcoPlates system consists of 31 different sole carbon sources plus a non-C control contained in 96-well microtiter plates (Garland, 1996; Insam and Goberna, 2004). Briefly, the plates were incubated at 26°C, and the optical density was read in a Microplate Reader at 590 nm after incubation for 24, 48, 96, and 120 h. The 48 h absorbance data were used for the analysis, as this was the time necessary for the microbial growth and color development. The substrate diversity calculated by the average-well color development index (AWCD) and the richness index (R) was estimated after an incubation time 48 h.

### Statistical Analysis

One-way analysis of variance (ANOVA) and comparison of means based on Tukey’s test were used to determine significant differences between the samples. Principal components analysis was also used to analyze the BIOLOG data to asses overall differences in the CLPP profiles of the soils and casts. Statistica Software was used to make statistical calculations.

## Results

### Enzymatic Activity

**FIGURE 1:**
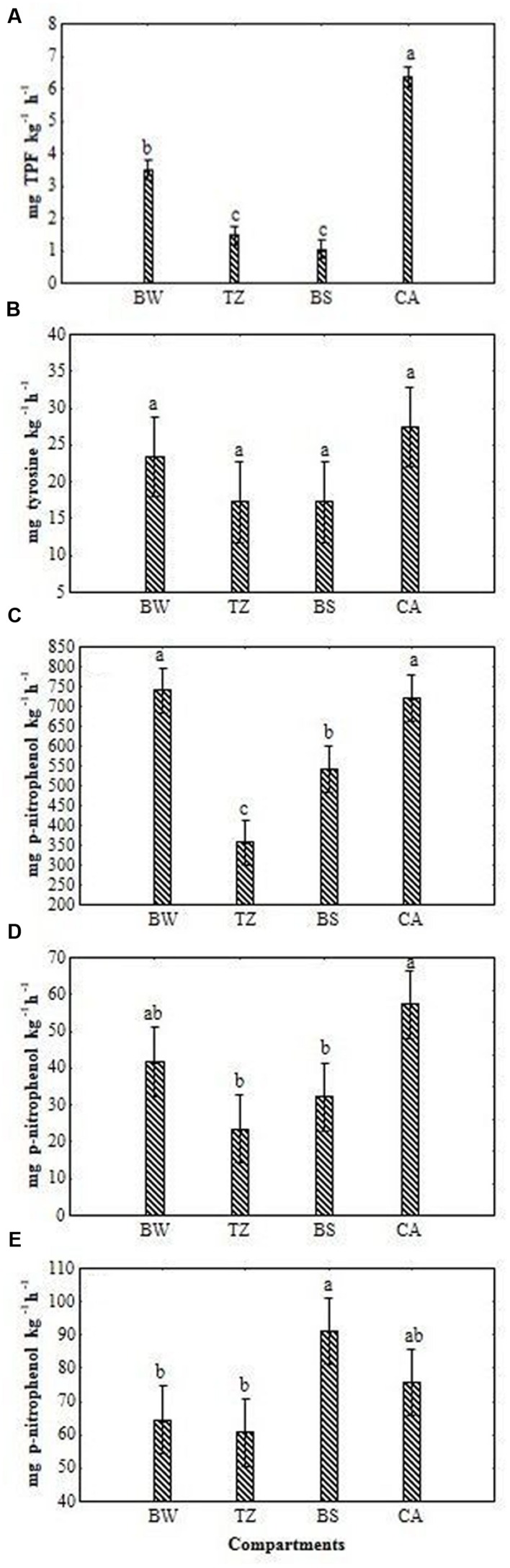
**Activities of dehydrogenase **(A)**, protease **(B)**, β-glucosidase **(C)**, alkaline phosphomonoesterase **(D)** and acid phosphomonoesterase **(E)** for different compartments of burrow system (BW, burrow wall; TZ, transitional zone; BS, bulk soil; CA, cast aggregates).** Error bars represent 0.95 confidence intervals. The different letters indicate significant differences between the compartments at *P* < 0.05.

All enzymatic activities except for acid phosphomonoesterase activity were on average the highest in BW and CA and the lowest in TZ and BS although the extent of the differentiation was related to the type of the activity (**Figures 1A–E**). In the case of dehydrogenase activity the lowest value in BS (3.50 mg kg^-1^ h^-1^) increased significantly (*P* < 0.05) in TZ, BW and CA by 41, 235, and 509%, respectively. The values of the protease activity were almost the same in BS and TZ (17 mg tyrosine kg^-1^ h^-1^) and increased (although not significantly) in BW (by 35%) and CA (by 58%). The β-glucosidase and alkaline phosphomonoesterase activity had the lowest respective values in TZ (356 and 24 mg *p*-nitrophenol kg^-1^ h^-1^), but they increased in CA and BW by 137–102% for the former and by 75–107% for the latter. The activity of acid phosphomonoesterase, however, was the largest in BS but not significantly different from that noted in the other compartments.

### Soil Microbial Functional Diversity and PCA Analysis

**FIGURE 2:**
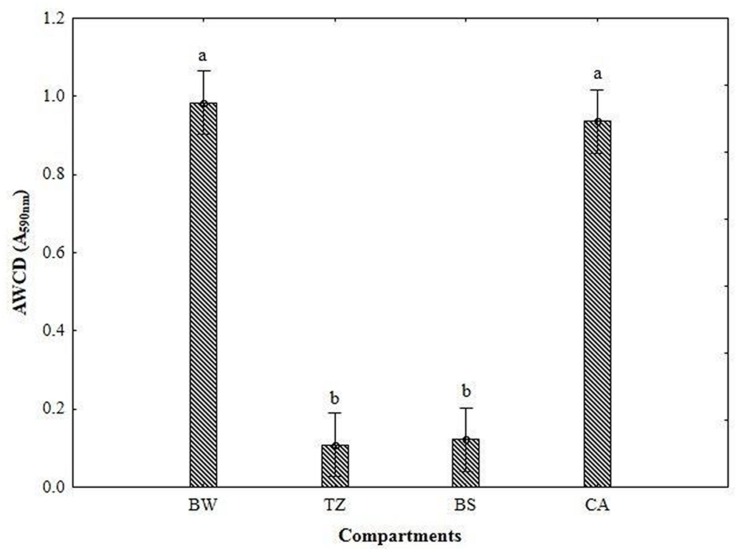
**Average well-color development (AWCD) of metabolized substrates calculated from Biolog data for different compartments of burrow system (BW, burrow wall; TZ, transitional zone; BS, bulk soil; CA, cast aggregates).** Error bars represent 0.95 confidence intervals. The different letters indicate significant differences between the compartments at *P* < 0.05.

**FIGURE 3:**
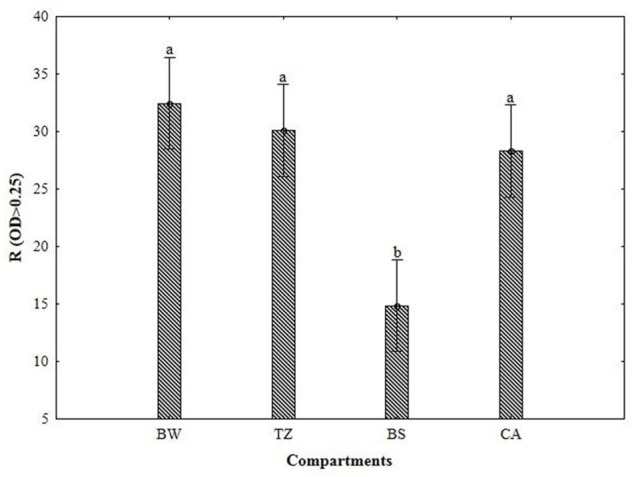
**Richness index (R) of metabolized substrates calculated from Biolog data for different compartments of burrow system (BW, burrow wall; TZ, transitional zone; BS, bulk soil; CA, cast aggregates).** Error bars represent 0.95 confidence intervals. The different letters indicate significant differences between the compartments at *P* < 0.05.

As can be seen from **Figure 2**, the values of the AWCD were substantially higher in BW and CA (0.98–0.94 A_590 nm_) than in TZ and BS (0.11–0.12 A_590 nm_). The richness index (**Figure 3**) was the lowest BS (>20 mm; 0.11–0.12 A_590 nm_) and considerably higher (by 86–113%) in the other compartments.

**FIGURE 4:**
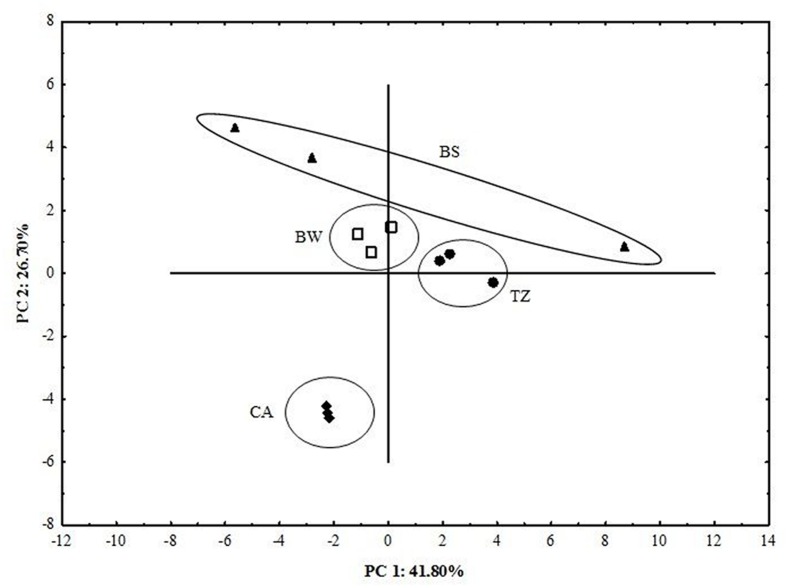
**Principal components analysis (PCA loadings) on variables data of carbon sources activity from the BW, TZ, BS, and CA**.

**FIGURE 5:**
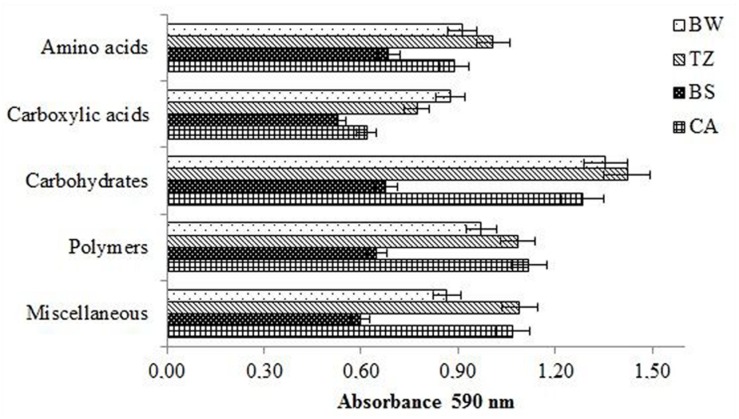
**Categorized substrates utilization pattern by microbial communities.** Errors bars represent the standard error of the mean (*n* = 3).

The principal component analysis (PCA) of the Biolog system data clearly distinguished the samples (**Figure 4**; **Table 1**). The first two principal components (PC1 and PC2) in PCA analysis explained 68.5% of the total variance in the CLPP dataset based on the soil and cast samples. Fifteen C sources were selected by this analysis as the most important in terms of the overall changes in the patterns of substrate utilization by the microbial populations of BW and casts. Values of scores of each C sources with PC1 and PC2 are shown in **Table 1.** The PC1 mainly differentiated the BW and TZ, which shifted positively along this function. Thus, the activity of soil collected from BW and TZ led to an increase in the potential microbial consumption of carbohydrates (D-Xylose and i-Erythriol), carboxylic acids (D-Glucosaminic Acid, 2-Hydroxy Benzoic Acid, 4-Hydroxy Benzoic Acid, Hydroxybutyric Acid, Itaconic Acid, α-Ketobutyric Acid), and amino acids (L-Arginine, L-Phenylalanine, L-Serine, L-Threonine, Glycyl-L-Glutamic Acid, Phenylethylamine; **Figure 5**; **Table 1**).

**Table 1:** Carbon substrates utilized by microorganisms in Biolog EcoPlate TM, significantly correlated to PC1 and PC2 (*R* > 0.70).

PC 1		PC2	
D-Xylose	0.810	Glycogen	-0.906
i-Erythritol	0.935	D-Cellobiose	-0.805
D-Glucosaminic Acid	0.933	β-Methyl-D-Glucoside	-0.938
2-Hydroksy Benzoic Acid	0.900	*N*-Acetyl-D-Glucosamine	-0.926
4-Hydroksy Benzoic Acid	0.932	DL-α-Glycerol Phosphate	-0.863
Hydroksybutiric Acid	0.820	D-Malic Acid	-0.883
Itaconic Acid	0.941	Putrescine	-0.873
α-Ketobutiric Acid	0.848		
L-Arginine	0.910		
L-Phenylalamine	0.884		
L-Serine	0.874		
L-Threonine	0.883		
Glycyl-L-Glutamic Acid	0.867		
Phenylethylamine	0.833		


The PC2 explained 26% of the total variance and contributed to differentiating the casts from the other compartments, mainly due to higher potential utilization of the polymers and other carbon sources (miscellaneous; **Figure 5**). It is worth noting that utilization of all categorized substrates was the lowest in the BS.

## Discussion

### Enzymatic Activities

Our results showed that all enzymatic activities except for acid phosphomonoesterase were substantially higher in BW and CA than in BS and TZ. The higher levels of the microbial activities in the earthworm-built structures can result partly from the greater quantity of total organic C and greater contribution of more easily degradable (labile) organic substances (i.e., the mucus) as indicated by the greater C_mic_:C_org_ ratios (Lipiec et al., 2015). The greater quantity of the organic substances in the BWs can be due to casting activity and grass residues as well as leaf litter transported by earthworms from the surface orchard sward. Using isotope C tracers, Andriuzzi et al. (2016) revealed that incorporation of labeled organic material was greater by approximately 16% in a drilosphere of grassed soil occupied by an anecic *Lumbricus centralis* (Bouché, 1972) than unoccupied drilosphere and attributed this to mobilization of soluble litter and casting activity. Furthermore, stimulation of physicochemical modification as well as breakdown and fragmentation of the organic matter in the drilosphere environment ensures a greater surface available for microorganisms enhancing enzymatic activity (Tiunov and Scheu, 1999). However, there were not substantial differences in substrate quality between the compartments as indicated by similar C to N ratios shown in an earlier study in the pear orchard (Lipiec et al., 2015).

The enzymatic activities in our study were not similarly, affected in all compartments. The dehydrogenase activity levels exhibited the greatest sensitivity to variable soil conditions in the studied compartments. Large increases in dehydrogenase activity in CA and BW (up to six times) compared to undigested soil in BS indicate that earthworms and their active microbiomes catalyze metabolic reactions, producing adenosine triphosphate through the oxidation of organic matter. The higher protease activity in the earthworm built structures than in the BS, related to emerging from the hydrolysis of protein N, indicates a larger pool of available dissolved organic N (Paul and Clark, 1996; Schimel and Bennett, 2004). The increase in the β-glucosidase activity in the structures implies greater potential for the turnover of carbon. In the study of Floch et al. (2009), β-glucosidase in apple orchard calcareous soils exhibited lower activity under organic management and higher activity under conventional and integrated pest management strategies compared to control.

Alkaline and acid phosphomonoesterase activities are linked to the P cycle, as they catalyze the release of inorganic phosphorus (orthophosphate) from organic phosphomonoesters (Alef and Nannipieri, 1995). In our study the alkaline phosphomonoesterase levels were the highest in the drilosphere environment and acid phosphomonoesterase in the BS. This increase in alkaline phosphomonoesterase might have been caused by the increase in microbial biomass (Lipiec et al., 2015) and the associated decrease in the content of phosphorus stimulating soil phosphatases (Doan et al., 2013). An additional explanation could be that part of alkaline phosphatases are already produced in the worm gut with a more effective priming effect of phosphorus that still remains in freshly released cast deposition (e.g., Le Bayon and Binet, 2006). However, the increase in acid phosphatase activity in the BS might have been caused by lower microbial biomass (Lipiec et al., 2015) and greater acidity compared to that in the gut and fresh deposits of earthworms (Lavelle et al., 1995). These results suggest that earthworms‘ cast can selectively and variously affect the enzymatic activity, depending on type of enzyme and soil characteristics in the soil (earthworm) compartments.

The response of the dehydrogenase activity in the present study is in line with the findings of Jégou et al. (2001) that the dehydrogenase activity compared to that of alkaline phosphatase and acid phosphatase showed the most striking differences between the earthworm affected compartments and BS. Since dehydrogenase activity together with microbial biomass give information on the total activity of the microbial community (Natal-da-Luz et al., 2012), the studied compartments can be lined up as CA > BW > TZ > BS.

The greater enzyme activity in this study along with the greater microbial biomass as shown in an earlier study (Lipiec et al., 2015) in earthworm influenced compartments vs. BS, indicates beneficial earthworm-microorganism interaction though microorganisms are major constituents of earthworms diet (Aira et al., 2007; Zirbes et al., 2012). Furthermore, these enhanced rates of enzyme activities in fresh deposits can be associated with both fragmentation and moistening of the organic resources and microbial development, already in earthworm gut. This explanation can be supported by the results of Egert et al. (2004) who using the terminal restriction fragment length polymorphism method revealed only small differences between bacterial and archaeal communities in earthworm gut and fresh casts. Conversely, BS accumulates organic material due to slow turnover rates (Rumpel and Kögel-Knabner, 2011; Kuzyakov and Blagodatskaya, 2015). Additionally, there was no mutual relationship between earthworms and microbial activity in composted soil (Doan et al., 2013) and during vermicomposting of residues (Hassina et al., 2014), which was attributed to competition between bacteria and earthworms for organic resources and/or to the consumption of microbes by earthworms.

### Linking Microbial Enzymatic Activities and Functional Diversity

Besides the changes in the enzyme activity, earthworms increased the AWCD and richness index and favored utilization of amino acids, carboxylic acids, carbohydrates, polymers, and miscellaneous compounds in the earthworm built structures compared to BS, as shown by carbon substrate consumption measured with the Biolog Ecoplate. The greater utilization of amino acids such as in the earthworm structures indirectly supports enhanced protease activity. Additionally the Biolog system allowed identifying an increase in the potential utilization of -Arginine, L-Phenylamine, L-Serine, L-Threonine, which significantly correlated to PC1 (separating BW and TZ), and L-Putrescine, which significantly correlated to PC2 (separating CA and BS). The similarly, greater utilization of D-Xylose and Glycogen, which significantly correlated to PC1 and PC2, respectively, correspond with the increased β-glucosidase activity.

Overall, the earthworm enhanced production of enzymes catalyzing metabolic reactions, hydrolysis of protein N, and turnover of carbon and P cycle associated with the AWCD, and consumption of specific carbon sources The responses can be associated with microbial succession that is linked to the changes in decomposition of soil organic matter (Nannipieri et al., 2003; Insam and Goberna, 2004).

### Microbial Activity in Relation to the Stability of the Orchard Ecosystem

It is worth noting that the mutual interactions between earthworms and microorganisms in the orchard litter-soil environment can be enhanced by abundance of earthworms and other invertebrates as well as high contribution of the rhizosphere structures in the grassed soil providing additional organic matter (or labile C) and forming microbial hotspots (Chamberlain et al., 2006; Kuzyakov and Blagodatskaya, 2015). As a consequence, a majority of microbial processes in the soil can take place in the hotspots. Enhanced microbial biomass and activity in the hotspots make soils less vulnerable to degradation and thus increase the stability of the ecosystem through buffering functional shifts of ecologically relevant processes induced by environmental deviations (Chen et al., 2015; Mendes et al., 2015).

Accelerated enzyme activity and functional diversity in the hotspots was observed in our study for freshly produced casts in the earthworm-occupied drilosphere and the surrounding soil surface. Such activity requires additional nutrients (e.g., N and P), causing their microbial mining from soil organic matter, i.e., priming effects that are consequences of hot moments with a high input of labile C and energy, temporarily removing the limitation common for BS (Görres et al., 2001; Kuzyakov and Blagodatskaya, 2015). However, the microbial abundance in the drilosphere declined in abandoned burrows due to the diminished enzyme activities and associated C turnover (Don et al., 2008). Recently, Andriuzzi et al. (2016) have found that also a population of eukaryotic protists and nematodes decreased in burrows after they had been abandoned by the anecic earthworm *L. centralis* (Bouché, 1972).

The results from the present study on microbial activity of the drilosphere environment agree well with earlier results indicating that earthworm burrows serve as preferential paths for vertical flow of water and agricultural chemicals and root growth (Lipiec and Hatano, 2003) and on the other hand, impede horizontal flow to the adjacent soil due to reduced pore size (Görres et al., 2001; Jégou et al., 2001) and wettability (Lipiec et al., 2015) as well as increased density (Rogasik et al., 2014). Further, earthworm casts deposited on the soil surface become stable aggregates after aging and are fundamental for minimizing microbial decomposition of carbon and soil crusting, erosion and compaction (Blanchart et al., 2004a; Lavelle, 2011; Jouquet et al., 2012), additionally, they influence water movement and gas diffusion (Capowiez et al., 2006; Alaoui et al., 2011). Thus, the biological and physical functions of the drilosphere and casts influence several ecological processes at the local (e.g., burrow) and landscape scales and enhance and conserve soil quality.

## Conclusion

1. Burrow walls and casts built by earthworms compared to the BS or TZ in the pear orchard displayed greater activities of enzymes including dehydrogenase, β-glucosidase protease, and alkaline phosphatase whereas the activity of acid phosphatase was the greatest in the BS. The dehydrogenase activity levels exhibited the greatest sensitivity to variable soil conditions in the studied compartments.
2. The earthworm-built structures, compared to BS, in general exhibited greater functional diversity of the microbial community as shown by the average-well color development and richness index. The greater enzymatic activities and the functional diversity in the earthworm-built structures were attributed to greater total organic carbon and contribution of labile carbon.
3. The principal components analysis of the data from Biolog EcoPlate clearly distinguished the compartments. The PC1 mainly differentiated the BWs and TZ, which shifted positively along this function and the PC2 differentiated the casts and the other compartments.
4. Overall, the presence of the earthworms promotes the maintenance of different enzymatic activities and catabolic potential in BWs and CAs compared to BS, and thus contributes to spatial diversity of microbial processes in grassed orchard soil.

## Author Contributions

Conceived and designed the experiments: JL, MF, and MB. Performed the experiments: MF, MB, MT, and KO.

## Conflict of Interest Statement

The authors declare that the research was conducted in the absence of any commercial or financial relationships that could be construed as a potential conflict of interest.
